# A Novel Labeling Approach Identifies Three Stability Levels of Acetylcholine Receptors in the Mouse Neuromuscular Junction *In Vivo*

**DOI:** 10.1371/journal.pone.0020524

**Published:** 2011-06-02

**Authors:** Siegfried Strack, Yvonne Petersen, Anika Wagner, Ira V. Röder, Marina Albrizio, Markus Reischl, Irene U. Wacker, Christoph Wilhelm, Rüdiger Rudolf

**Affiliations:** 1 Institut für Toxikologie und Genetik, Karlsruhe Institute of Technology, Karlsruhe, Germany; 2 Institut für Angewandte Informatik, Karlsruhe Institute of Technology, Karlsruhe, Germany; 3 Institut für Biologische Grenzflächen, Karlsruhe Institute of Technology, Karlsruhe, Germany; 4 Sicherheitsmanagement Analytische Labore, Karlsruhe Institute of Technology, Karlsruhe, Germany; Claremont Colleges, United States of America

## Abstract

**Background:**

The turnover of acetylcholine receptors at the neuromuscular junction is regulated in an activity-dependent manner. Upon denervation and under various other pathological conditions, receptor half-life is decreased.

**Methodology/Principal Findings:**

We demonstrate a novel approach to follow the kinetics of acetylcholine receptor lifetimes upon pulse labeling of mouse muscles with ^125^I-α-bungarotoxin *in vivo*. In contrast to previous assays where residual activity was measured *ex vivo*, in our setup the same animals are used throughout the whole measurement period, thereby permitting a dramatic reduction of animal numbers at increased data quality. We identified three stability levels of acetylcholine receptors depending on the presence or absence of innervation: one pool of receptors with a long half-life of ∼13 days, a second with an intermediate half-life of ∼8 days, and a third with a short half-life of ∼1 day. Data were highly reproducible from animal to animal and followed simple exponential terms. The principal outcomes of these measurements were reproduced by an optical pulse-labeling assay introduced recently.

**Conclusions/Significance:**

A novel assay to determine kinetics of acetylcholine receptor turnover with small animal numbers is presented. Our data show that nerve activity acts on muscle acetylcholine receptor stability by at least two different means, one shifting receptor lifetime from short to intermediate and another, which further increases receptor stability to a long lifetime. We hypothesize on possible molecular mechanisms.

## Introduction

Muscle contraction in vertebrates is mediated by the activation of nicotinic acetylcholine receptors (AChRs) at the nerve-muscle synapse, the neuromuscular junction (NMJ). NMJs exhibit a peculiar gross-morphology and ultrastructure [1], [2], [3]. En face view of NMJs reveals elaborate structures, which often assume a roundish, circular to ellipsoid shape with diameters of roughly 20–50 µm. Internally, these shapes consist of AChR-rich band-like arbors, which have a width of about 1–3 µm and are usually all connected with each other, forming what is often called a “pretzel” appearance. Notably, pre- and postsynaptic arbors of the pretzel normally overlap perfectly, indicating an intense and fine-tuned crosstalk that mediates proper positioning of components on both sides. Postsynaptically each arbor exhibits a rich membrane topology with secondary clefts branching off from the primary cleft, which itself connects to the smooth presynaptic membrane. The crests of folds created by the secondary clefts are covered with roughly 10,000 AChRs per µm^2^. Another hallmark of the postsynaptic apparatus is the presence of some 4–7 subsynaptic or fundamental nuclei, which lie directly beneath the postsynaptic membrane and which, in the muscular syncytium, specialize on the production of synapse-specific transcripts, such as AChR, acetylcholine esterase or rapsyn [4]. While NMJs are built in the unborn animal and are then kept in place for very long time periods, AChRs have lifetimes of only several days [1].

In order to maintain the high density of AChRs at the junctional crests and to permit the perfect matching to the presynapse over time, AChR transport mechanisms are required [1]. Exo- and endocytic processes mediate delivery of newly formed and retrieval of old/excess receptors, respectively. In addition, recycling of AChRs commences in the first postnatal days in mouse and adapts AChR density in the postsynapse to strain [5], [6], [7]. Furthermore, this process may be necessary for restructuring purposes. AChR recycling is linked to metabolic stabilization of AChRs, which in turn is highly dependent on the activity of motor nerve and muscle [5], [6], [8], [9], [10], [11]. While in healthy innervated mouse muscles receptor half-life is ∼10–13 days, denervation reduces the receptor half-life to ∼1 day [8], [12]. Since muscle stimulation [13] as well as pharmacologically induced increase in cAMP levels [14], [15] recovers AChRs in denervated muscles from fast turnover, muscle-dependent as well as nerve-dependent processes seem to modulate receptor stability. At the postsynaptic side myosin Va forms a complex with recycling AChRs and PKA RIα and the cooperative action of this complex is important for proper recycling of AChRs [9], [10], [11], [16].

To address AChR turnover kinetics, different approaches have been adopted so far. Harnessing the quasi-irreversible binding of the snake venom, α-bungarotoxin (BGT) [17] and the possibility to link BGT to biotin, fluorescent dyes or radioisotopes, microscopic and scintillation counting-based methods were established. While microscopic approaches have mainly delivered important insights into the spatial and structure-function aspects of AChR turnover [5], [6], [7], [8], [9], [10], [16], [18], radioactive assays using ^125^I-BGT have revealed the basic quantitative insights into the time constants underlying AChR turnover [12]. Albeit being the method of choice for determining precise AChR kinetics, a widespread use of the radiolabeling technique also for genetically manipulated mouse strains might have been hampered by the large quantities of animals and radioisotope necessary to achieve reliable statistics. We here introduce a novel ^125^I-BGT-based assay to determine AChR turnover, which allows reducing the number of animals and the starting material by a factor of at least ten without compromising data quality. Furthermore, we describe the presence of three levels of AChR stabilization and discuss possible mechanisms underlying the formation of these receptor pools.

## Results

### A portable detector system for the study of AChR lifetime kinetics

**Figure 1:**
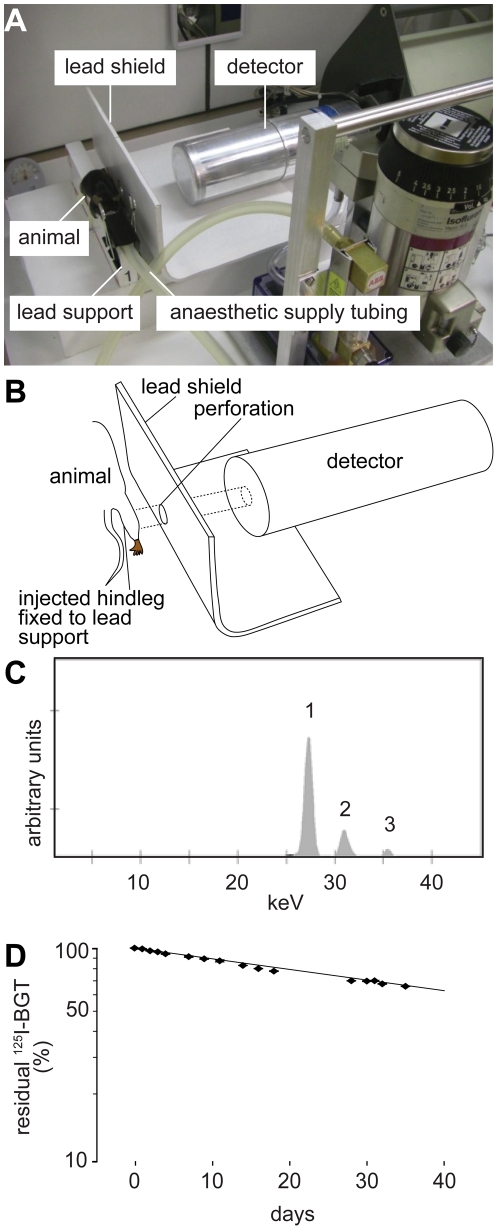
A novel setup allows monitoring of AChR turnover in live mice. A: Photograph of the setup showing the anaesthetized ^125^I-BGT-injected mouse mounted onto the lead support shielding left from right body half. A lead shield with a 16.5 mm wide perforation at the height of the TA muscle limits the detected ^125^I-emission to that of the lower hindlimb. B: Schematic drawing illustrating the emission path from injected lower hindlimb to the Germanium semiconductor sensor. C: Typical emission spectrum as detected upon injection of ^125^I-BGT. The integrals below peak 1 were used for quantification of AChR turnover. D: Decay of a non-injected ^125^I-BGT-standard. Dots, measured values; dotted line, exponential fit based on measured values; continuous line, theoretical decay curve of ^125^I.

In this study we established an assay to follow lifetime kinetics of AChRs using *in vivo* pulse labeling of AChRs with ^125^I-BGT and subsequent repetitive measurement of ^125^I emission with a portable Germanium semiconductor counter. ^125^I-BGT was injected once into the tibialis anterior (TA) muscles of anaesthetized mice, which were then mounted onto a lead support (Fig. 1A). To measure ^125^I emission only from the injected muscle and not from the whole animal, a lead shield with a circular fenestration at the height of the mounted TA muscle was placed between mouse and detector (Fig. 1A–B). In most cases, both legs were injected and during denervation experiments (see below) one side served as untreated control. For each leg, a measurement period of five minutes was sufficient. By using such short detection windows together with volatile isoflurane anesthesia (Fig. 1A) the entire measurement series could be performed using identical animals. Fig. 1C shows a typical irradiation spectrum as detected by the sensor and Fig. S1 depicts the precise setup of the detector. Measurement of a standard ^125^I-BGT-injection dose kept in the test tube as a reference revealed a t_1/2_ of 58.7 days for ^125^I (Fig. 1D). This was very similar to the theoretical t_1/2_ of this nuclide of 59.4 days (Decay Data Evaluation Project, http://www.nucleide.org/DDEP_WG/DDEPdata.htm), and shows that even much longer t_1/2_ than the ones expected for AChRs (about 10 days) could be measured reliably using this approach.

**Figure 2:**
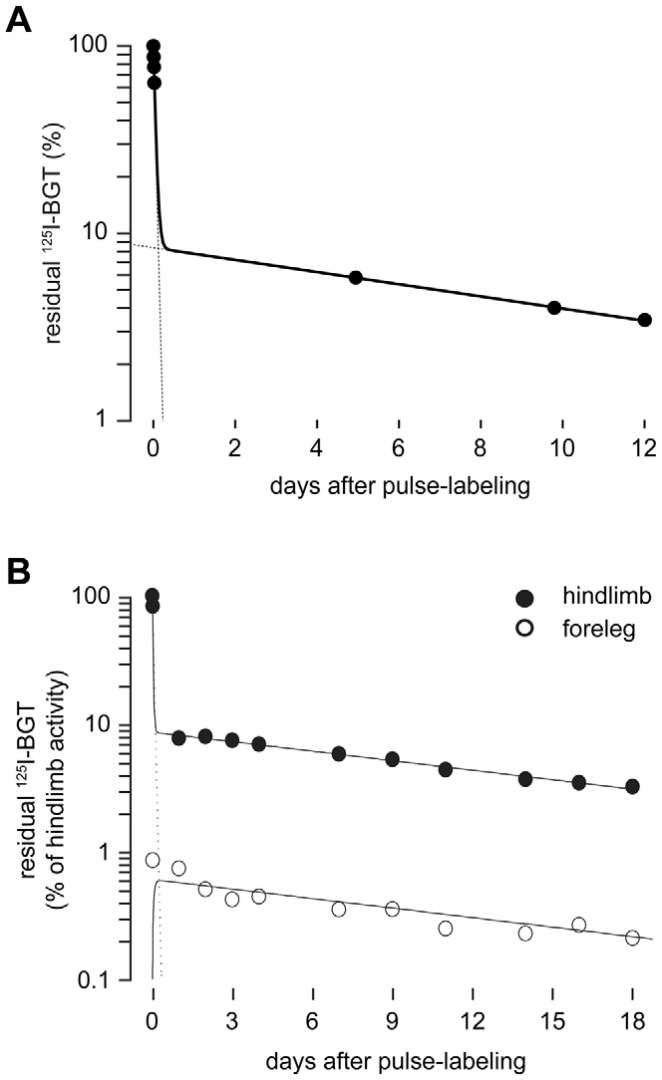
Most intramuscularly injected ^125^I-activity rapidly becomes available systemically. ^125^I-BGT was injected into TA muscles of mice. ^125^I-emission was repetitively measured immediately after injection (time point 0) and subsequently at time points as indicated. A: Residual ^125^I-BGT-emission in the injected hindlimb as a function of time after pulse labeling. Dots, measured values; continuous line, two-term exponential fit; dotted lines, extrapolations for the two different exponential terms. This data series had four measurements shortly after ^125^I-BGT injection. A t_1/2_ of 49 min was calculated for the expulsion of ^125^I-activity from the injected TA muscle during the first day after pulse labeling. B: ^125^I-BGT-emission in the injected hindlimb (black dots) and the ispilateral foreleg (white dots) normalized to the value measured immeditaley after pulse labeling in the injected hindlimb as a function of time after pulse labeling. Continuous lines show a two-term exponential fit and a ‘Bateman’ function for the hindlimb and the foreleg, respectively. The latter describes the result of the two exponential processes, (i) infiltration of ^125^I-BGT upon release from the hindlimb and (ii) subsequent exponential decay with the same half-life as in the hindlimb. The dotted line indicates the extrapolation for the fit of the expulsion component. Note, that systemically available ^125^I-BGT accumulates in foreleg muscles and shows a similar decay as in the injected hindlimb muscle.

**Figure 3:**
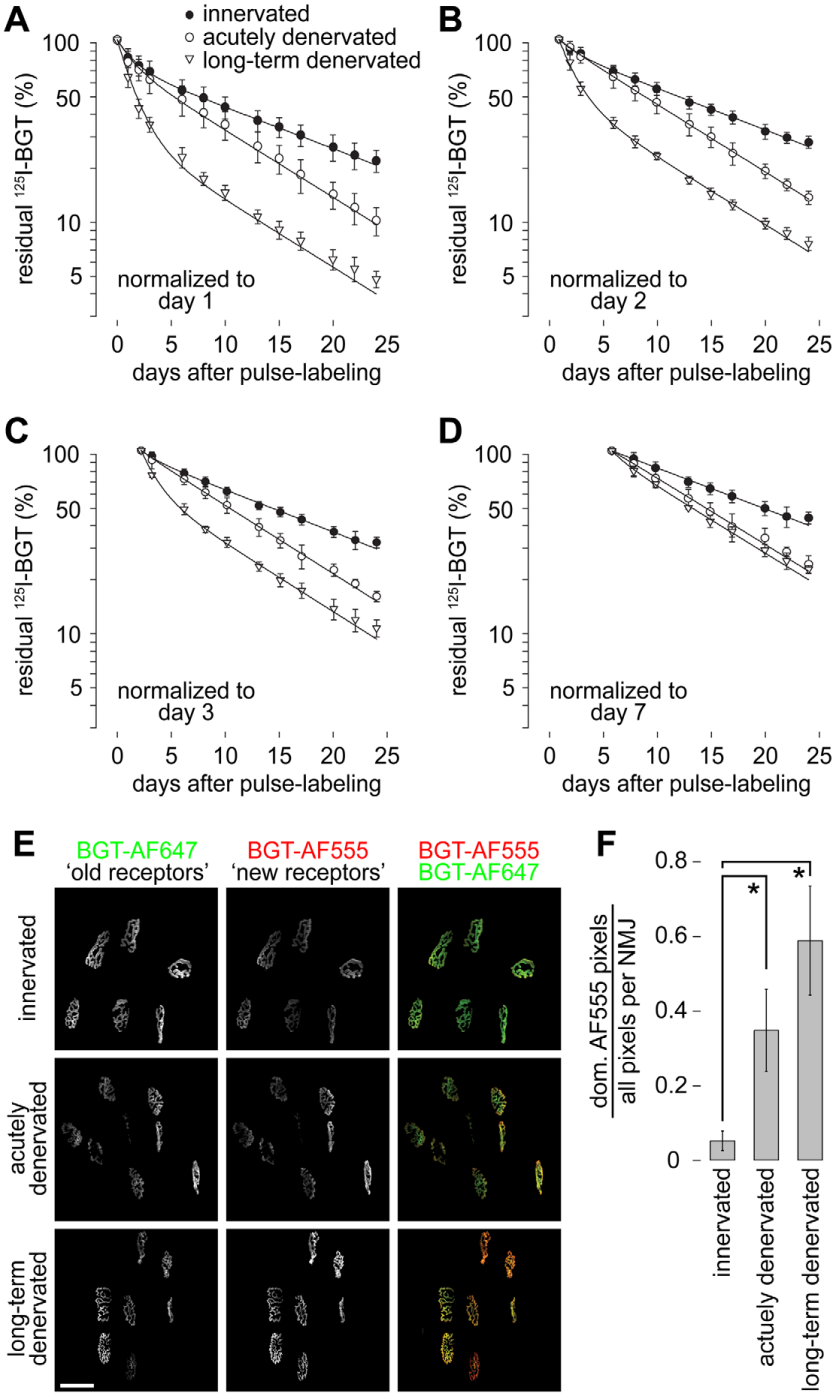
The novel approach reveals three levels of metabolic stabilization of AChRs. A–D: Muscles were either left innervated (innervated) or the were denervated immediatedly prior to (acutely denervated) or five days before pulse labeling with ^125^I-BGT (long-term denervated). Time point of pulse labeling is t0. Graphs in A–D show the residual ^125^I-emission in the injected hindlimbs as a function of time after pulse labeling. Dots, measured values (mean ± SD; n-values: control n = 10, acutely denervated n = 5, long-term denervated n = 4); lines, two-term exponential fits. Data are normalized to the mean values measured on day 1 (A), day 2 (B), day 3 (C) or day 7 (D) to highlight the progressive loss of the short-lived AChR population with increasing time after pulse labeling. During the first week of chase muscles were measured at intervals of 24 hours. Already at the day 1 measurement less than 10^−5^% of unbound ^125^I-BGT was left. E–F: Muscles were innervated or denervated as in A–D, but instead of ^125^I-BGT BGT-AF647 was injected at t0. Ten days later BGT-AF555 was injected to label newly formed AChRs. Subsequently, *in vivo* imaging was performed and synapses were automatically segmented and analyzed. E: Representative maximum z-projections of microscopic fields showing signals of BGT-AF647 (‘old receptors’), BGT-AF555 (‘new receptors’) and overlay of both (‘old receptors in green’, ‘new receptors’ in red) on day ten after labeling with BGT-AF647. Scale bar, 50 µm. F: Quantification of the fraction of pixels with dominant ‘new receptor’ label of the entire NMJ pixels as a function of innervation/denervation status of the analyzed muscle. Mean ± SEM (n-values: control n = 3, acutely denervated n = 4, long-term denervated n = 3). * P<0.05 according to Welch test.

Based on these findings, we started first *in vivo* experiments to determine the distribution of intramuscularly injected ^125^I-BGT within the body. Repetitive measurements in the first minutes after injection showed a rapid loss of radioactive signal from the injected muscles (Fig. 2A shows a representative trace). This suggested that most of the injected ^125^I-BGT did not bind to AChRs but quickly left the TA muscle, presumably through the blood stream or the lymphatic system. Due to this process one day after ^125^I-BGT-injection about 90% of the initially measured radioactivity was lost from the injected muscles. From that time point onwards, the decline of ^125^I-BGT-emission was much slower and roughly followed a single exponential term in innervated mice (Fig. 2A, for more detailed analyses, see Fig. 3) with a ^125^I-half-life-corrected decay time of about 11 days (Fig. 2A). These values are very similar to previously described results [12]. To better understand the destination of the residual ^125^I-BGT, we measured the amounts of ^125^I after a 25 days long experiment in mouse and litter. Cage litter was sampled on days 1, 7 and 25 after pulse labeling to investigate the kinetic properties of probe expulsion. As shown in Table 1, about 60% of the injected ^125^I was excreted during the first day after injection. In the following period, the amount of radioactivity present in the litter samples progressively decreased, adding up to a total of 70% of the total radioactivity in the litter. Five percent of the total activity was found in the animal on day 25 after pulse labeling, leaving a loss of 25%. We speculate that most of this activity was present in feces and urine released during the measurement periods, in particular, during the first measurement and during the following wake-up period. As to the activity residing in the animal over the entire experimental period, we expected most of it to enrich in other skeletal muscles. To investigate this, we analyzed the forelegs of hindlimb-injected mice. Interestingly, about 1% of the total injected ^125^I-BGT was detected there and exhibited a very similar, roughly single-exponential decay as that in the TA muscle (Fig. 2B). This suggests that ^125^I-BGT released during the first minutes after injection is accumulating in other muscles, where it binds to AChRs. The similarity in the decay times of both observed muscles indicates that the amount of ^125^I-BGT injected into the TA muscle did not significantly interfere with AChR turnover at the site of injection.

**Table 1:** Most ^125^I activity is excreted by the animal during the first day after pulse labeling.

Sample	^125^I activity(% of total injected activity*)
litter day 1 after pulse labeling	60
litter days 2–7 after pulse labeling	9
litter days 8–25 after pulse labeling	1
mouse	5
loss	25

TA muscles of an anaesthetized mouse were injected with ^125^I-BGT on day zero. The cage litter was sampled on days 1, 7, and 25 after pulse labeling. Litter samples and the mouse after sacrificing on day 25 after pulse labeling were measured for ^125^I activity. Data are from a representative experiment. *All values were corrected for their radioactive decay.

### Dependence of AChR lifetime on different muscle-activity stimuli is revealed by two independent approaches

Next, we injected left and right TA muscles of anesthetized mice with ^125^I-BGT and simultaneously denervated the left legs by cutting the sciatic nerves on this side (acutely denervated in Fig. 3). Contralateral muscles were kept innervated. In another set of experiments, one side was denervated five days prior to pulse labeling (long-term denervated in Fig. 3) and the contralateral muscle was again kept innervated as control. Since all control values were very similar, they were pooled in the graphs depicted in Fig. 3. Subsequent repetitive monitoring of ^125^I emission over a time period of four weeks showed interesting features. First, data sets obtained under all three experimental conditions could be nicely fitted with double-exponential terms (Fig. 3A), indicating the presence of two distinct AChR populations in each preparation. However, while two AChR populations were found in all cases, population sizes and their durations were dependent on the specific condition. An overview on these values is given in Table 2. We found that a fast decaying component with a t_1/2_ of 1.1±0.4 days (mean ± SEM) was present in all three preparations. This is easily visible upon comparing Fig. 3A–D, showing that the apparent size of this compartment was dependent on the starting point of normalization. Indeed, upon normalization of the data set to day seven after pulse labeling (Fig. 3D) the fast compartment was undetectable in innervated and acutely denervated muscles. Furthermore, while this population was largest in the long-term denervated muscles it was present at similar amounts in innervated and acutely denervated specimens. When concentrating on the last three weeks of data sampling (see e.g. Fig. 3D), it was evident that two different slowly decaying AChR populations existed. AChRs from innervated muscles were more stable than those from denervated ones. This was reflected by half-lives of 13.1 days and 8.1 days for innervated and denervated muscles, respectively. The slight offset in the long-term denervated curve in Fig. 3D is due to a little remnant of the fast component in these muscles. Altogether, these data show the presence of three different types of receptor stability, i.e. high, intermediate and low.

**Table 2:** AChRs exhibit three different half-lives, depending on the state of innervation.

	compartment size%	rate constant(d^−1^)	half-life(d)	mean lifetime (d)
innervatedfast compartment	13±2	0.630±0.191	1.1±0.3	1.6±0.5
innervatedslow compartment	87±2	0.053±0.002	13.1±0.4	18.9±0.6
acutely denervatedfast compartment	5±2	0.630±0.417	1.1±0.7	1.6±1.1
acutely denervatedslow compartment	95±2	0.086±0.002	8.1±0.2	11.6±0.2
long-term denervatedfast compartment	51±4	0.630±0.070	1.1±0.1	1.6±0.1
long-term denervatedslow compartment	49±4	0.086±0.007	8.1±0.6	11.6±0.6

TA muscles of anaesthetized mice were injected with ^125^I-BGT on day zero and then ^125^I emission from injected hindlimbs was measured over 25 days. Regressions from data sets normalized to day 2 after pulse labeling were calculated using two-exponential terms and t_1/2_ values of 1.1 days (fast compartment), 8.1 days (intermediate compartment) and 13.1 days (slow compartment). The table shows compartment sizes, rate constants, half-lives, and mean receptor lifetimes, all ± SD (n-values: control n = 10, acutely denervated n = 5, long-term denervated n = 4).

To further consolidate these findings, we used a recently described in vivo imaging approach [9], [10], where two distinct AChR pools are differentially color-labeled. Therefore, in a first round, mice were unilaterally denervated (acutely denervated) by cutting the left sciatic nerve and the preexisting AChRs were simultaneously marked on both legs with BGT coupled to AlexaFluor 647 (BGT-AF647). Ten days later, the same muscles were again injected with BGT, which was now labeled with AF555. Subsequent in vivo imaging revealed the relative distributions of AF647 (old receptors) and AF555 signal intensities (new receptors) (Fig. 3E). Quantitative analysis of the fraction of pixels with dominant AF555 signals per NMJ showed a significant ratio increase in denervated muscles, indicating a shorter AChR lifetime under this condition (Fig. 3F). In a second series of experiments, nerves were cut five days before the injection of BGT-AF647 (long-term denervated). Again ten days later, NMJs were imaged after acute labeling of new AChRs with BGT-AF555 (Fig. 3E). This showed an even higher increase of the AF555/AF647 ratio (Fig. 3F), suggesting that old receptors labeled in a denervated muscle underwent a further reduction of their lifetime.

## Discussion

The earliest attempts to determine the activity-dependent lifetime of synaptic AChRs date back to the 1970’s [19] and scintillation counter-based assays using ^125^I-BGT as a labeling probe were optimized and widely used up to the 1990’s [12], [20]. At this time the principal role of nerve activity on metabolic stabilization of AChRs was completely established. However, the rather recent finding of considerable functional analogies between the NMJ and central synapses [11], [21], including activity-dependent neurotransmitter receptor recycling [5], [6], [7], [8], [16], the role of receptor-associated proteins [22], and the role of PKA-dependent signaling and class V myosins for regulating receptor turnover [9], [10], [11], have triggered novel interest in this branch of NMJ research. Furthermore, the demonstration of the beneficial effect of long-term caloric restriction on NMJ structure preservation in aged mice [23] are likely to place the NMJ center stage between body metabolism and muscle performance in aging and metabolic diseases. Based on these aspects and the ongoing production of relevant genetic mouse models in these areas of research there is need for efficient tools to study AChR turnover as a major diagnostic feature of NMJ integrity and NMJ activity. The method presented here of quantitatively determining AChR turnover shares the use of ^125^I-BGT with previous assays. However, a special Germanium semiconductor sensor in this study allows measuring AChR decay kinetics in situ. Therefore single mice can be used for entire measurement series without compromising data quality. This reduces the amount of animals needed to acquire reliable data by a factor of 10–15 compared to previous settings. This is particularly relevant for the investigation of rare or weakly reproducing strains.

Similar to previous reports [14], [15], [24] our studies using innervated and denervated conditions have revealed three different receptor lifetimes with t_1/2_ values of 1.1 day, 8.1 days and 13.1 days. However, in contrast to previous measurements the new in vivo assay permits the acquisition of complete kinetics for individual mice, thereby reducing the uncertainty of biological variability. The presence of all three populations was highly reproducible and the lifetime values of the different populations were very similar to previous reports [8], [14], [15], [24]. Notably, the short-lived population was found to be present under all experimental conditions, i.e. also under innervation. Very likely, these short-lived receptors represent extrasynaptic AChRs, which are formed at low density all along the muscle fiber. An alternative explanation would be that transient blockage of synapse activity by bungarotoxin binding to AChRs led to increased receptor turnover [8]. However, to achieve such inactivation more than 70% of receptors need to be occupied by bungarotoxin [25]. We estimate to label only about 40% of AChRs in the current approach, a value that was also used by others as a non-blocking dose of bungarotoxin [8]. We do therefore not expect any effect of the labeling procedure on receptor stability. This is supported by the finding, that forelegs, which took up small amounts of the bungarotoxin injected into the hindlimbs, showed similar AChR lifetime kinetics (Fig. 2B) as well as by the observation that the animals did not drag their feet upon injection. Nonetheless, the fact, that we can not distinguish clearly between those different localizations of AChRs is an obvious caveat of the current approach. The size of the short-lived pool was similar in innervated and acutely denervated muscles, confirming previous studies which showed that destabilization of AChRs to the short-lived kinetics of about one day is not an immediate process, but involves incorporation of novel receptors [15], [16]. This was further corroborated by our long-term denervation experiment: there, most AChRs were replaced by short-lived receptors. In summary, these data corroborate earlier studies in that nerve-activity-dependent signals are necessary to slow down AChR turnover beyond the basal, 1-day half-life.

**Figure 4:**
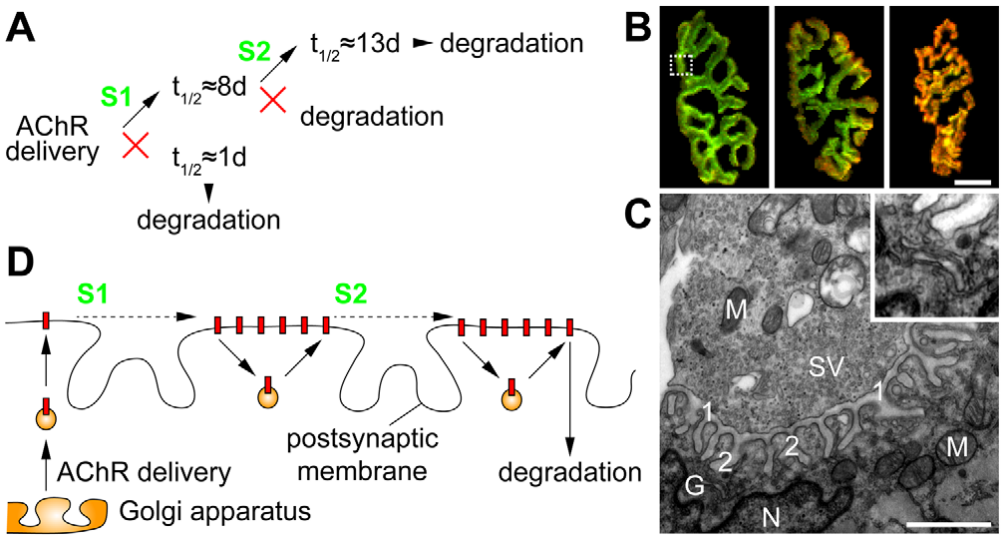
Two nerve-activity-dependent signals mediate full metabolic stabilization of AChRs. A: Scheme summarizing the findings of the ^125^I-BGT approach. Upon delivery of AChRs to the synapse, the presence of the nerve-activity-dependent signal, S1, triggers the prolongation of the receptor half-life from ∼1 to ∼8 days. When not stabilized receptors are degraded. During or at the end of the first week another nerve-activity-dependent signal, S2, is needed to extend the receptor half-life to now ∼13 days. B: Enlargements from Fig. 3E indicate the preferential incorporation of newly formed AChRs at the periphery of NMJs. Pictures show NMJs of innervated (left), acutely denervated (middle) or long-term denervated (right) muscles. ‘Old receptors’ are shown in green, ‘new receptors’ in red. Note, that with increasing time of denervation-induced absence of AChR stabilization the domain where ‘new receptors’ prevail progressively expands from the periphery of NMJs towards the center. Scale bar, 10 µm. C: Transmission electron micrograph through a NMJ. The picture shows the cross-section of one arbor (exemplified by the dotted rectangle in B, left panel). The upper left half shows the presynaptic terminal, the lower right half shows the muscular, post-synaptic apparatus. 1, primary synaptic cleft; 2, secondary synaptic cleft; G, Golgi apparatus; M, mitochondria; N, subsynaptic nucleus; SV, synaptic vesicles. Scale bar, 1 µm. The region of the Golgi apparatus (for better visibility contrast-corrected) is enlarged in the insert. D: Scheme illustrating the hypothesized lateral movement of AChRs within the NMJ and the presumptive spatial localization of S1 and S2. Red rectangles, AChRs; spheres, exocytic (left) and recycling vesicles (middle and right).

Soon after denervation transcription is switched from adult (α_2_βεδ) to embryonic-type AChRs (α_2_βγδ) [16]. Since a similar switch, just from embryonic to adult AChRs, occurs in the early postnatal phase [26], when receptor stabilization is taking place in mice [16], it is intriguing to speculate that the γ-to-ε switch could be the molecular mechanism underlying the changes in activity-dependent AChR lifetimes. However, a recent study has shown that perinatal AChR stabilization is not depending on ε-subunits [7] rendering unlikely that a transcription-dependent mechanism is adopted upon denervation. Thus, other processes like competition for space at the synapse [7] or posttranslational modifications of AChRs or of accompanying molecules can be envisaged to account for shifting the short to a longer half-life. In the following discussion we call this unknown mechanism “signal 1” (S1, Fig. 4).

The comparison of denervated and innervated muscles revealed a further step of AChR stabilization shifting the t_1/2_ of the long-lived AChR population from 8 days in the denervated to 13 days in the innervated condition. Similar observations were made earlier [24], [27]. This implies the existence of a second signal (S2, see Fig. 4A), which is to be present between days two and eight after receptor incorporation. Thus, a two-step activity-dependent signaling appears to be necessary to fully stabilize AChRs (Fig. 4A). Previous studies from different labs have shown that AChR distribution within NMJs is unequal, showing higher densities at the borders than at the center of synapses [1]. Furthermore, also the addition of newly synthesized AChRs occurs in an asymmetric fashion [9], [16], [18]: Being added at the periphery of the synapse, they appear to travel towards the center of NMJs with increasing lifetime (Fig. 4B). Electron microscopy of individual arbors of the NMJ gross morphology (Fig. 4C) shows that subsynaptic nuclei, which are specialized in the production of synapse-specific transcripts, are in close proximity to the folded postsynaptic apparatus. The secretory pathway, through which AChRs have to funnel to reach the synaptic membrane, is weakly developed around subsynaptic nuclei, suggesting a limited transcriptional activity of transmembrane proteins. Notably, Golgi apparatuses are often found on one end of synaptic branches as shown in Fig. 4C (see label “G”) adding morphological support to the observation of asymmetric receptor incorporation. However, if AChRs are not added evenly to the synapse, they must be distributed subsequently by other mechanisms. We speculate that this is achieved either by directed lateral transport along the plasma membrane or by directed recycling processes. In both cases the net flux of AChRs would be from the lateral side of incorporation to the central region (Fig. 4D). Overlaying AChR lifetimes and the hypothetical process of AChR movement within the postsynaptic apparatus during this lifetime, we come up with a concluding hypothesis. According to this hypothesis the two steps of nerve-dependent receptor stabilization are executed at different sites of the NMJ. We think that the first signal, S1, is likely to reach the receptors at or close to the site of receptor incorporation into the synapse, i.e. at the rim of the NMJ (Fig. 4D). Subsequent directed movement of the AChRs allows the receptors to reach the site of the secondary stabilizing signal, S2, which would then be located more towards the center of the synapse (Fig. 4D).

Many aspects of this hypothetical model still need to be solved. These include the precise location of S1 and S2, the mode of lateral translocation of AChRs, the origin and nature of S1 and S2, and how S1 and S2 induce stabilization of AChRs. Finally, it would be very interesting to know, if additional signals would be able to further prolong receptor lifetime, or if a t_1/2_ of about 13 days is just the maximum obtainable.

## Materials and Methods

### Ethics statement

Use and care of animals was as approved by German authorities (Tierschutzkommission of the Regierungspräsidium Karlsruhe, license G-22/07) according to national law (TierSchG §7).

### Chemicals

Lyophilized radioactive α-BGT[^125^I]Tyr54 (^125^I-BGT) with a specific activity of 81.4 TBq/mmol (2200 Ci/mmol) was from Perking Elmer. Injection-ready solution was prepared by adding sterile aqua dest. according to manufacturer description. Decay of ^125^I is mainly characterized by electron capture (93%). The whole spectrum is showing X-ray-, electron- and gamma peaks. BGT coupled to AlexaFluors 555 (BGT-AF555) and 647 (BGT-AF647) were from Invitrogen.

### Animals, anaesthesia, denervation and ^125^I-BGT injection

For experiments, 20–32 weeks old male wild-type mice were bred in our rodent facility. Denervation was performed as described previously [10] and labeling of TA muscles was done under anesthesia with a combination of Xylazin (Bayer) and Zoletil (Laboratories Virbac) by intraperitoneal injection. During measurements of ^125^I-BGT animals were anaesthetized with an Isoflurane – air mixture (0.6–1.5%) administered by a tube for about 10 minutes. For hindlimb denervation 5 mm of the sciatic nerve were removed. Success of denervation was checked in sacrificed mice at the end of each experiment. Right hindlimbs of each mouse served as innervated control. For labeling muscle AChRs 10 µl of ^125^I-BGT, containing 0.46 MBq (2,5 µCi) ^125^I, were injected into dissected TA muscles in both hindlimbs. In long-term denervation experiments ^125^I-BGT was injected five days after denervation. A single dose of ^125^I-BGT averaged to 0.01 µg or 1 pmol. LD_50_ of α-BGT is about 3.5 µg per 100 g body weight [28].

### Measurements of ^125^I emission *in vivo*

For *in vivo* detection of emitted X-rays from ^125^I-BGT-labeled TA muscles a portable Germanium semiconductor counter (Model GX3018, Canberra, Belgium) was used. Anaesthetized mice were mounted onto a lead support and both hindlimbs were fixed in a defined position for detection. The lead support of 20 mm thickness efficiently blocked radiation cross talk between both legs during measurement. To further narrow down emission detection to the hindlimbs, radiation deriving from the whole mouse body was blocked by a lead shield with a circular fenestration for the TA muscle (Fig. 1A–B). Duration of each measurement for a single leg was 300 s (statistical counting error <2%). Stability of the detector system and the chosen geometry was checked by repeated measurements of an aliquot of ^125^I-BGT (10 µl) in a test tube. Data from the detector were analyzed using an attached multi channel analyzer (InSpector 2000 DSP Portable Spectroscopy Workstation, Canberra). In all experiments we used the X-ray peak area in the range between 25.5 to 28.5 keV (peaks at 27.2 keV and 27.5 keV). All experiments were performed under an extractor hood.

### Measurements of ^125^I emission *ex vivo* and of litter samples

For an approximate assessment of radioactivity distribution during the experiments, single mice were kept in separate cages. Residual radioactivity in the litter and the mouse was measured at the end of the experiment using stationary Germanium semiconductor counters in an ILAC (www.ilac.org) on ISO 17025:2005 accredited lab.

### Data analysis

Experimental data of AChR degradation were assessed first visually and subsequently numerically using SigmaPlot (Systat software, Inc.). Exponential release of ^125^I-BGT was analyzed by regression equations with two exponential terms (using four parameters) and least square fits using the following term:
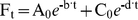
(1)To analyze the receptor bound kinetics data were typically normalized to day two after pulse labeling as described elsewhere [15], when the unbound ^125^I-BGT was evacuated completely. All error bars represent standard error of the mean. Significance analysis of degradation rates used Student’s t-test. For characterization of exponentially first order processes several parameters were derived: 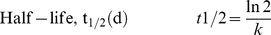
(2)

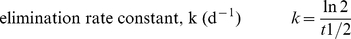
(3)

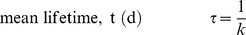
(4)


### *In vivo* imaging and data analysis

Microscopic determination of AChR turnover (Fig. 3E) and automated data extraction and analysis (Fig. 3F) were as described [10]. Briefly, 3D stacks at 512×512 pixel resolution were taken of BGT-AF647 (‘old receptors’) and of BGT-AF555 signals (‘new receptors’) using a 63x objective and confocal in vivo imaging. Then, synapses were automatically identified and segmented in 3D using a custom-made algorithm [10]. For each pixel in the segmented NMJs the signal intensity values for AF555 and AF647 were extracted. Then, the fraction of pixels per NMJ where the AF555 signal intensity was higher than that of AF647 was calculated.

### Electron microscopy

Mice were killed by cervical dislocation, TA muscles were removed and fixed overnight at 4°C in 2% paraformaldehyde/1.25% glutaraldehyde in 0.1 M Pipes pH 7.0 containing 0.1% NaN_3_. Postfixation for 1 h on ice in 0.5% OsOs_4_/ 0.8% K_3_[Fe(CN)_6_] in 0.1 M Pipes was followed by block-staining overnight at 4°C in 2% uranylacetate/25% ethanol. Samples were dehydrated in ethanol and infiltrated via propylenoxide with Epon according to standard protocols.

## Supporting Information

Figure S1 **Schematic of the detector setup.** All values indicate distances in millimeters. A: Top view of the detector setup. B: Side view of the detector setup. C: Side view of the lead block used to mount the anesthetized animal. The red ring indicates the position, where hindlimb muscles were fixed.
(TIF)
