# Supplementary figures and images for: A Novel Labeling Approach Identifies Three Stability Levels of Acetylcholine Receptors in the Mouse Neuromuscular Junction In Vivo
Source: PLoS One. 2011 Jun 2;6(6):e20524. doi: 10.1371/journal.pone.0020524 (PMC3107218; doi:10.1371/journal.pone.0020524)

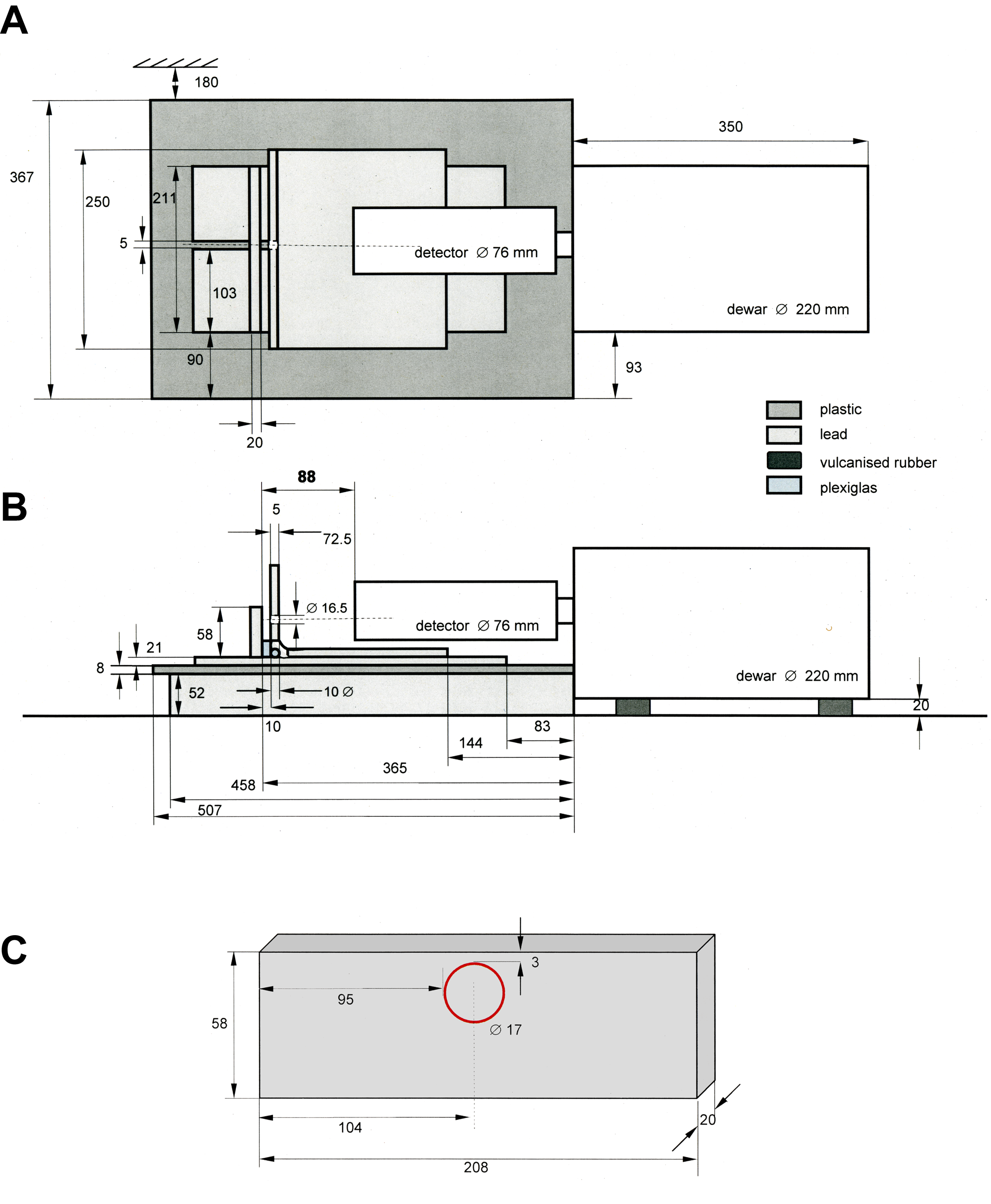

Supplement: Figure S1 — Schematic of the detector setup. All values indicate distances in millimeters. A: Top view of the detector setup. B: Side view of the detector setup. C: Side view of the lead block used to mount the anesthetized animal. The red ring indicates the position, where hindlimb muscles were fixed. (TIF) [file pone.0020524.s001.tif]
